# Laboratory Studies of Feeding and Oviposition Preference, Developmental Performance, and Survival of the Predatory Beetle, *Sasajiscymnus tsugae* on Diets of the Woolly Adelgids, *Adelges tsugae* and *Adelges piceae*

**DOI:** 10.1673/031.011.6801

**Published:** 2011-05-26

**Authors:** Robert M. Jetton, John F. Monahan, Fred P. Hain

**Affiliations:** ^1^ Department of Entomology, N.C. State University, Raleigh, NC, 27695, USA; ^2^ Department of Statistics, N.C. State University, Raleigh, NC, 27695, USA; ^3^ Current address: Camcore, Department of Forestry & Environmental Resources, N.C. State University, Raleigh, NC, 27695, USA

**Keywords:** *Abies fraseri*, alternate rearing host, balsam woolly adelgid, biological control, hemlock woolly adelgid, prey suitability, *Tsuga canadensis*

## Abstract

The suitability of the balsam woolly adelgid, *Adelges piceae* Ratzeburg (Hemiptera: Adelgidae) as an alternate mass rearing host for the adelgid predator, *Sasajiscymnus tsugae* Sasaji and McClure (Coleoptera: Coccinellidae) was studied in the laboratory. This predator is native to Japan and has been introduced to eastern hemlock, *Tsuga canadensis* (L.) Carrière (Pinales: Pinaceae), forests throughout the eastern United States for biological control of the hemlock woolly adelgid, *Adelges tsugae* Annand (Hemiptera: Adelgidae), also of Japanese origin. Feeding, oviposition, immature development, and adult long-term survival of *S. tsugae* were tested in a series of no choice (single-prey) and paired-choice experiments between the primary host prey, *A. tsugae*, and the alternate host prey, *A. piceae*. In paired-choice feeding tests, the predator did not discriminate between eggs of the two adelgid species, but in the no choice tests the predator did eat significantly more eggs of *A. piceae* than those of *A. tsugae*. *S. tsugae* accepted both test prey for oviposition and preferred to lay eggs on adelgid infested versus noninfested host plants. Overall oviposition rates were very low (< 1 egg per predator female) in the oviposition preference tests. Predator immature development rates did not differ between the two test prey, but only 60% of *S. tsugae* survived egg to adult development when fed *A. piceae* compared to 86% when fed *A. tsugae*. *S. tsugae* adult long-term survival was significantly influenced (positively and negatively) by prey type and the availability of a supplemental food source (diluted honey) when offered aestivating *A. tsugae* sistens nymphs or ovipositing aestivosistens *A. piceae* adults, but not when offered ovipositing *A. tsugae* sistens adults. These results suggest that the development of *S. tsugae* laboratory colonies reared on a diet consisting only of *A. piceae* may be possible, and that the biological control potential of the predator might be expanded to include management of *A. piceae* in Christmas tree plantations.

## Introduction

The hemlock woolly adelgid, *Adelges tsugae* Annand (Hemiptera: Adelgidae), is an important forest pest in the eastern United States that has become a serious threat to eastern hemlock, *Tsuga candensis* (L.) Carrière, and Carolina hemlock, *T. caroliniana* Engelmann (Pinales: Pinaceae), ecosystems throughout Appalachian Mountain region. Since its introduction to Richmond, Virginia on imported hemlock nursery stock in 1951 (Stoetzel 2002), *A. tsugae* has spread to 18 eastern states from New England to Georgia, where it infests approximately 50% of hemlock ecosystems and can kill trees in as little as four years (McClure et al. 2003). The adelgid is thought to be of Japanese origin (Annand 1924; Havill et al. 2006) where it is relatively harmless to the native *Tsuga* spp. due to a combination of host resistance and natural enemies (McClure et al. 2003). On the hemlock hosts of eastern North America, *A. tsugae* has a complex polymorphic and parthenogenetic life cycle with two generations per year called the sistens (over-wintering generation), present from July–March, and the progrediens (spring generation), present from March–June (McClure 1989). A third winged sexual generation called the sexuparae also occurs, but lacks a suitable host in North America (McClure 1987).

Logistic, economic, and ecological concerns over the use of insecticides in forest settings have focused *A. tsugae* management efforts on biological control. Due to a lack of effective native or naturalized adelgid predators in the eastern United States (Wallace and Hain 2000), emphasis has been placed on a classical biological control approach (Cheah et al. 2004). There are no known parasitoids of the Adelgidae, but a number of promising predators of *A. tsugae* have been identified and imported for quarantine evaluation (Cheah et al. 2004). Several of these have been approved for free release into eastern forests, but to date only one, *Sasajiscymnus* (formerly *Pseudoscymnus*) *tsugae* Sasaji and McClure (Coleoptera: Coccinellidae), has been successfully mass reared and widely distributed. As of 2007, approximately 3.5 million *S. tsugae* had been released in 16 states reporting hemlock infestations by *A. tsugae* (Salom et al. 2008). Laboratory and field studies revealed that the predator is well suited for biological control of *A. tsugae*; it feeds preferentially on, and has a life cycle well synchronized with, the adelgid and overwinters in the hemlock habitat (Cheah and McClure 1998, 2000). Under natural conditions, *S. tsugae* has two generations per year that overlap with those of *A. tsugae*, each developing through 4 larval instars, prepupal, and pupal stages before emerging as an adult (Cheah and McClure 2000).

*S. tsugae* produced in mass rearing facilities are reared on a diet of live *A. tsugae* collected from branches with naturally occurring infestations. Given the large number of beetles already released into hemlock stands it is clear that these mass rearing programs have been successful. However, in the field, the *A. tsugae* sistens generation enters an aestival diapause lasting from mid July through October (McClure 1987), placing an important constraint on predator production (Palmer and Sheppard 2002). The aestivating adelgid nymphs available for colony feeding during this period constitute a less nutritious food source for *S. tsugae* and negatively influence survival rates and female egg production (Palmer and Sheppard 2002). This results in a three to four month period during which mass rearing stocks decline, with no new predators produced or released. *A. tsugae* aestival diapause is thought to be maternally regulated and temperature dependent (Salom et al. 2001), but it is not clear if these cues can be manipulated to prevent the induction of diapause and avoid the *S. tsugae* mass rearing delays associated with the discontinuous supply of suitable prey material.

The use of alternate rearing hosts that are active during *A. tsugae's* aestival diapause may provide a means to overcome this constraint on *S. tsugae* mass rearing programs. One candidate prey with potential is the balsam woolly adelgid, *Adelges piceae* Ratzeburg (Hemiptera: Adelgidae), another serious exotic forest pest in North America that attacks true firs (*Abies* spp.) and has eliminated approximately 95% of mature Fraser fir, *Abies fraseri* (Pursh) Poiret (Pinales: Pinaceae), from the high elevation spruce-fir forest type in the southern Appalachian Mountains (Dull et al. 1988; Mitchell and Buffam 2001; Jenkins 2003). Like *A. tsugae, A. piceae* has a complex polymorphic life cycle consisting of two to four generations per year (Balch 1952). The first is called the hiemosistens (over-wintering generation) and is present from September–June. Subsequent generations are called aestivosistens (spring and summer generations) and are present from June–September. The *A. piceae* aestivosistens generation is actively feeding, developing, and reproducing during the same period that the *A. tsugae* sistens generation is in diapause (Balch 1952; McClure 1989). Thus, it may be possible to augment or substitute *A. piceae* for *A. tsugae* as the main prey item in *S. tsugae* mass rearing facilities during the late summer months to maintain colony survival and beetle production, providing a year round supply of beetles for *A. tsugae* biological control.

The objective of this study was to determine through a series of laboratory evaluations if *A. piceae* might be suitable as an alternate rearing host for *S. tsugae* during the aestival diapause of *A. tsugae*. The predator's feeding and ovipositional preference, developmental performance, and adult survival on *A. piceae* versus *A. tsugae* were tested in a series of no choice (single prey) and paired- choice bioassays. The effect of a supplemental food source (diluted honey) alone and in combination with adelgid prey on *S. tsugae* survival was also evaluated.

## Materials and Methods

### Source of Predators and Test Prey

*S. tsugae* eggs and adults were obtained from mass rearing colonies maintained at the N.C. State University (NCSU) Insectary reared under protocols developed at the New Jersey Department of Agriculture's Phillip Alampi Beneficial Insect Laboratory at Trenton, NJ (Palmer and Sheppard 2001). The NCSU colony was developed from small starter colonies of 100 beetles (50♂:50♀) obtained from the Alampi Lab and the North Carolina Department of Agriculture Beneficial Insect Laboratory (Cary, NC). All eggs used were < 24 hours old and adults were > 1 month and < 6 months old and reared the same year as experimentation.

The test adelgid prey were obtained from naturally occurring populations in Ashe and Avery Counties of North Carolina. The primary host prey, *A. tsugae*, was collected by cutting adelgid infested eastern hemlock branches and placing them in buckets with water. Branches were collected in March for tests with ovipositing sistens adults, and in August for tests with aestivating sistens nymphs. The secondary host prey, *A. piceae*, was collected by felling infested Fraser fir trees in abandoned Christmas tree plantations. Felled trees were limbed, cut into 1 m bolts, and then each bolt was set upright in a bucket of moist sand and the top end was sealed with paraffin wax to maintain hydration. Bolts were collected in March for tests with ovipositing adults of the hiemosistens generation, and in August for tests with the aestivosistens generation. Both test prey were held in a rearing room at the NCSU Insectary at 16° C, 50% RH, and 12:12 L:D. The various life stages of predators and test prey used in each experiment are summarized in Table 1.

### Test Arenas

All tests of *S. tsugae* feeding and oviposition preference and developmental performance between *A. tsugae* and *A. piceae* were conducted using the same basic experimental design. Experimental units were 9 × 2 cm polystyrene Petri dishes with a 1.5 cm diameter ventilation hole covered with a 0.6 mm fabric mesh. Each dish was lined with a single layer of filter paper (Whatman No. 1) that was moistened with a methylparaben solution (0.50 g/250 ml distilled water) to inhibit fungal growth, and a 2 cm piece of dental cotton wick moistened with distilled water was provided as a water source for *S. tsugae*. Adelgid prey were presented intact on their host plant and consisted of 5 cm long *A. tsugae* infested eastern hemlock twigs and 2 cm diameter *A. piceae* infested bark rounds of Fraser fir. Bark rounds were extracted from fir bolts using a 2 cm diameter laboratory cork borer. All experiments were conducted in a 1700 Series Hotpack laboratory incubator (www.hotpack.com) at 26° C, 16:8 (L:D), and 70–80% RH, the same environmental conditions used for the mass rearing of *S. tsugae*.

All dishes were sealed with Parafilm during experimentation.

### Feeding Preference

The feeding preference of *S. tsugae* between *A. tsugae* and *A. piceae* eggs was evaluated in no choice (single-prey) and paired-choice experiments. The no choice test consisted of 40 Petri dishes split among the two prey treatments (*n*=20 dishes/adelgid species), each containing 50 eggs intact within woolly masses of the assigned test prey. In the paired-choice test, 50 eggs each of *A. tsugae* and *A. piceae* were placed together in petri dishes (n=20). Prior to each experiment all active, first instar adelgid crawlers were removed from host material and a single *S. tsugae* adult that had been starved for the preceding 12 hours was randomly assigned to each dish. Dishes were completely randomized in the incubator and predators were allowed to feed freely for 72 hours, after which the number of adelgid eggs consumed was recorded. This number was calculated via the following equation: eggs consumed = 50 - (eggs remaining + crawlers present). Because host material was cleared of all adelgid crawlers prior to these experiments, any crawlers present afterwards would have hatched from the 50 eggs placed in each dish and could not be considered consumed by *S. tsugae*. The volume of *A. tsugae* and *A. piceae* eggs was also estimated using an ocular micrometer fixed to the eyepiece of a stereoscope to measure the long and short axis of 100 eggs of each adelgid species. Egg volume in cubic micrometers was estimated based on the volume of a prolate spheroid via the following equation: adelgid egg volume = 4/3 πab^2^, where a is the length of the long axis and b is the length of the short axis.

### Oviposition Preference

The ovipositional preference of *S. tsugae* females for *A. tsugae* and *A. piceae* was evaluated in no choice (single-prey) and paired-choice experiments. The no choice test included 80 Petri dishes split among 4 treatments: *A. tsugae* infested eastern hemlock, *A. piceae* infested Fraser fir, noninfested eastern hemlock, and non-infested Fraser fir (*n* = 20 dishes/treatment). Each dish contained a single section of adelgid infested host plant with 10 (±2) woolly egg masses or non-infested host plant. In the paired-choice test, 10 (±2) egg masses of *A. tsugae* and *A. piceae* were paired together in petri dishes (*n* = 20). For both experiments, dishes were completely randomized in the incubator, and *S. tsugae* male-female pairs were randomly assigned to Petri dishes and allowed to feed, mate, and oviposit over a 72-hour period. After this time, the number of predator eggs laid was counted. Because *S. tsugae* eggs closely resemble the eggs of both adelgid prey species and females tend to lay eggs in concealed locations, all Petri dishes were held at experimental conditions for 10 days following completion of the test to rear out the predator larvae from eggs in order to verify egg counts. Both the number of *S. tsugae* eggs laid and larvae hatched were recorded.

### Developmental Performance

The suitability of *A. piceae* as a developmental host for *S. tsugae* from the egg to the adult stage was compared to that of *A. tsugae* in a no choice (single-prey) developmental performance test. The test included 30 Petri dishes split among the two prey treatments (*n* = 15 dishes/adelgid species). Using a fine brush, a single *S. tsugae* egg, < 24 hours old, was placed in each dish on the host plant section containing 10 (±2) woolly egg masses of the assigned test prey. Petri dishes were completely randomized in the incubator and examined daily for *S. tsugae* egg hatch, larval molt to the next life stage, and adult emergence. Fresh prey was added to dishes during each day's examination. Larval molt was signified by the presence of an exuvium, and the pre-pupal stage was determined to be when mature fourth instar larvae became sedentary and had a pronounced woolly covering. For each *S. tsugae* individual, the duration in days and survival to each life stage was recorded (1 = alive; 0 = dead).

### Adult Survival

Two experiments were conducted to evaluate survival of *S. tsugae* adults over a 36 day period using test prey alone or in combination with a supplemental food source. In survival test 1, the diet treatment combinations were *A. tsugae* plus food supplement, *A. tsugae* alone, *A. piceae* plus food supplement, *A. piceae* alone, food supplement alone, and a control (no test prey or food supplement). In survival test 2 the treatments were *A. tsugae* plus food supplement, *A. tsugae* alone, food supplement alone, and a control. *Adelges tsugae* was presented on 10 cm infested eastern hemlock twigs and *A. piceae* on 10 × 3 cm sections of infested Fraser fir bark (see Table 1 for insect life stages used in these experiments). The supplemental food source consisted of diluted honey (50:50 honey: distilled water) presented on 5 × 3 cm pieces of sterilized filter paper. Experimental units were 20 × 6 × 6 cm polystyrene rearing cages (Consolidated Plastics Co., Inc., www.consolidatedplastics.com) with a 2 cm diameter ventilation hole covered with a 0.6 mm fabric mesh. Each cage was lined with a double layer of paper towel (Georgia Pacific, www.gp.com) moistened with a methylparaben solution (0.50 g/250 ml distilled water) to inhibit fungal growth, and a 5 cm piece of dental cotton wick moistened with distilled water as a water source for *S. tsugae*. Plant sections with test prey were placed in the bottom of the cage and the filter paper with the honey supplement was attached to the sidewall. Each cage received five randomly assigned adult *S. tsugae* and were sealed with Parafilm (*n* = 5 boxes/diet treatment). The cages were examined once daily over the 36 day period of each test. During each day's observation the number of live adult beetles remaining in each cage was recorded as well as the location of each live beetle. Locations were recorded as on the host plant (hemlock twig or Fraser fir bark section), honey strip (feeding at the food supplement), water wick (drinking at the cotton water wick), or wandering about the test arena. Fresh test prey and food supplement was added every other day.

### Statistical Analysis

The no choice (single-prey) tests for feeding preference and developmental performance and egg volume estimates were analyzed using two sample *t* tests to determine the effect of prey type on *S. tsugae* adult feeding rate and the developmental time for each predator life stage. A paired *t* test was performed to determine adult *S. tsugae* prey preference in the paired-choice test for feeding preference. All *t* tests were performed using the Analyst Application in SAS 9.1 (SAS Institute 2003). A Chi-Square test (PROC FREQ, SAS 9.1) was performed to determine if the frequency of *S. tsugae* survival in each life stage was significantly different between prey types in the developmental performance test. Due to the overall low egg laying rates the oviposition preference data were not subjected to statistical analysis.

Logistic regression analyses were performed using the General Model Procedure (PROCGENMOD, SAS 9.1) to determine the probability of *S. tsugae* adult survival on day 36 when fed different diet treatment combinations in the adult survival tests. For survival test 1, the main effect of test prey type (*A. tsugae, A. piceae*, or none) on survival was tested for diet treatments with (*A. tsugae* + supplement; *A. piceae* + supplement; Supplement alone) or without (*A. tsugae; A. piceae*; control) the food supplement, and the likelihood estimates for these probabilities were calculated via the following formulae:
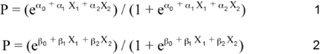

where P is the probability of *S. tsugae* adult survival; α_0_, α_1_, and α_2_ are regression coefficients with the food supplement present; β_0_, β_1_, and β_2_ are regression coefficients with the food supplement absent; X_1_ is an indicator variable denoting absence (0) or presence (1) of prey *A. tsugae;* and X_2_ is the indicator variable for prey *A. piceae*.

For survival test 2, the main effects of prey type (*A. tsugae* or none) and food supplement (present or absent) on *S. tsugae* adult survival were tested, and the likelihood estimates for this probability (P) was calculated via the following formula:
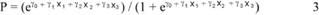

where P is the probability of *S. tsugae* adult survival; γ_0_ is the regression coefficient when both the food supplement and *A. tsugae* are absent; γ_1_,γ_2_,and γ_3_ are regression coefficients with the food supplement absent (γ_1_) or present (γ_2_ and γ_3_); and X_1_, X_2_, and X_3_ are indicator variables denoting absence (X_2_) or presence (X_1_ and X_3_) of *A. tsugae*.

## Results

### Feeding Preference

*Sasajiscymnus tsugae* adults consumed eggs of both test prey species. When given no choice (single-prey test), *S. tsugae* ate significantly more *A. piceae* than *A. tsugae* eggs (Table 2), indicating a preference for the former. However, in the paired-choice test, the predator demonstrated no feeding preference between *A. tsugae* eggs and those of *A. piceae*, consuming the same number of eggs of both prey (Table 3). Mean egg volume differed significantly between the test prey (*t* = 12.80, *df* = 198, *p* < 0.0001) and was greater for *A. piceae* (5205.53 µm^3^) compared to *A. tsugae* (3855.47 µm^3^).

### Oviposition Preference

*Sasajiscymnus tsugae* females found both test prey species to be acceptable for oviposition, but host plants alone were not suitable. In the no choice (single-prey) oviposition test, the predator demonstrated a clear preference for laying eggs on adelgid infested compared to non-infested host plant material (Table 4). Nor did *S. tsugae* females discriminate between *A. tsugae* infested hemlock or *A. piceae* infested Fraser fir as an oviposition substrate, laying similar numbers of eggs in close proximity to both test prey (Table 4). However, in the paired-choice experiment *S. tsugae* females appeared to prefer oviposition on *A. piceae* infested Fraser fir compared to *A. tsugae* infested hemlock (Table 5). In all test arenas, the number of larvae hatching several days after the completion of the oviposition trials exceeded the number of eggs counted.

### Developmental Performance

*Sasajiscymnus tsugae* was able to complete development to the adult stage on both test prey species (Table 6). The duration of the *S. tsugae* egg (*t* = 0.78, *df* = 28, *p* = 0.4380), instar 1 (*t* = 0.17, *df* = 28, *p* = 0.8597), instar 2 (*t* = 0.49, *df* = 23, *p* = 0.6252), instar 3 (*t* = 1.51, *df* = 22, *p* = 0.1449), instar 4 (*t* = 0.94, *df* = 21, *p* = 0.3545), pre-pupal (*t* = 0.24, *df* = 21, *p* = 0.8106), and pupal (*t* = 0.72, *df* = 20, *p* = 0.4773) life stages did not differ significantly between *A. tsugae* and *A. piceae*. The overall total developmental time to the adult stage for *S. tsugae* was slightly shorter on a diet of *A. piceae* compared with *A. tsugae*, but again, this difference was not significant (*t* = 0.51, *df* = 20, *p* = 0.6173). The percentage of *S. tsugae* individuals surviving to complete each life stage was lower for *A. piceae* compared to *A. tsugae* beginning with Instar 2, and was marginally significant at α = 0.10 for the pupal and adult stages (χ^2^ = 2.72, *df* = 1, *p* = 0.09).

### Adult Survival

**Figure 1.**
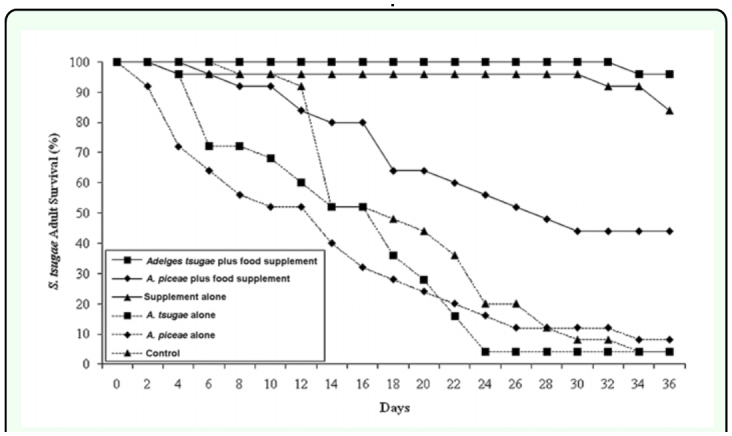
Adult *Sasajiscymnus tsugae* percent (%) survival over a 36 day period in long-term survival test I, conducted between August and September 2005 at 26° C, 16:8 (L:D), and 70–80% RH. High quality figures are available online.

Thirty-six day survival rates for *S. tsugae* adults were found to be influenced by both test prey species and the presence or absence of the supplemental food source. In survival test 1, the probability of *S. tsugae* adult survival on day 36 was significantly affected by the test prey (*A. tsugae, A. piceae*, or no prey) in diet treatment combinations that included the food supplement (χ^2^ = 7.38, *df* = 2, *p* = 0.0249). When the food supplement was available, the presence of *A. piceae* significantly reduced predator survival (χ^2^ = 7.51, *df* = 1, *p* = 0.0061) compared to the *A. tsugae* and supplement alone treatments (Figure 1). The probability of predator survival did not differ between *A. tsugae* + supplement and supplement alone (χ^2^ = 2.00, *df* = 1; *p* = 0.1573), although *S. tsugae* survival was slightly higher when both the prey (*A. tsugae*) and supplement were provided (Figure 1). Among treatments that did not include the food supplement (*A. tsugae, A. piceae*, or control), the probability of predator adult survival was not significantly affected by test prey type (χ^2^ = 0.59, *df* = 2, *p* = 0.7452) and survival in all three treatments was ≤ 8% (Figure 1).

**Figure 2.**
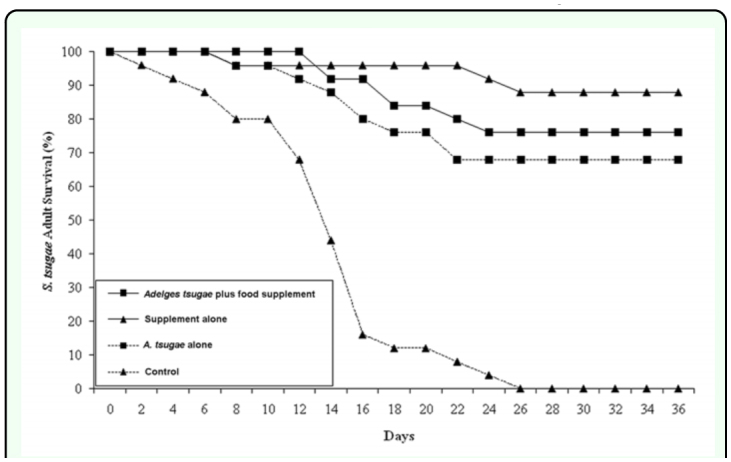
Adult *Sasajiscymnus tsugae* percent (%) survival over a 36 day period in long-term survival test 2, conducted between April and May 2006 at 26° C, 16:8 (L:D), and 70–80% RH. High quality figures are available online.

In survival test 2, the importance of the supplemental food source for the survival of *S. tsugae* adults was much reduced when the predators were provided a diet consisting of actively developing and ovipositing *A. tsugae* sistens adults (Figure 2). *Sasajiscymnus tsugae* survival was best in the supplement alone and *A. tsugae* + supplement treatments and lowest when predators we offered only *A. tsugae*. However, these trends were not significant and neither the test prey (χ ^2^ = 1.84, *df* = 1; *p* = 0.1753) nor the food supplement (χ ^2^, *df* = 1; *p* = 0.4292) affected the probability of *S. tsugae* adult survival on day 36. No predators survived to day 36 in the control treatment (no test prey, no food supplement).

The behavioral response of *S. tsugae* location within test arenas during each day's observation was heavily influenced by the test prey offered (Tables 7 and 8). In both survival tests 1 and 2, averaged over the entire 36 day period, 61–80% of adult beetles were found on the host plant in treatments with *A. tsugae* infested eastern hemlock twigs compared to 16–38% of beetles wandering about the test arenas. This trend was reversed for treatments with *A. piceae* infested Fraser fir bark in survival test 1 where *S. tsugae* appeared to prefer wandering about the test arena to being on the host plant. In all treatments that included the food supplement at least a few beetles were found feeding at the honey strip. As expected, in the supplement alone and control treatments the vast majority (93–99%) of *S. tsugae* were found wandering about the test arenas.

### Discussion

Prior to its widespread release in the eastern United States for biological control of *A. tsugae, S. tsugae* had been anecdotally reported to feed on other adelgid species (Cheah and McClure 1996), but its potential host range remained poorly defined. Laboratory studies conducted subsequent to widespread release demonstrated that while the predator has strong feeding preferences for most *A. tsugae* life stages, it will feed on the eggs, nymphs, and adults of adelgids in the *Adelges* and *Pineus* genera (Butin et al. 2004). In the laboratory preference and performance studies reported in the current study, *S. tsugae* accepted *A. piceae* for egg feeding, oviposition, and immature development and demonstrated little preference between *A. tsugae* and *A. piceae*.

In the feeding preference tests, *S. tsugae* did not discriminate between *A. tsugae* or *A. piceae* in paired-choice experiments, and consumed equal numbers of eggs of both adeglids. Similarly, Butin et al. 2004 found that the predator readily fed on the eggs of *A. laricis, A. cooleyi*, and *P. strobi*, preferring the eggs of *A. tsugae* only to those of *A. laricis*. Interestingly, however, in the current study *S*. *tsugae* did demonstrate a preference for the eggs of *A. piceae* over *A. tsugae* in the no choice (single-prey) test. Whether or not this result indicates a true feeding preference for *A. piceae* over *A. tsugae* by the predator remains unclear. The two adelgids feed on different parts of their respective host plants. *Adelges tsugae* is a xylem feeder on eastern hemlock (Young et al. 1995) while *A. piceae* feeds in the cortical parenchyma of Fraser fir outer bark (Balch 1952). This difference might influence the relative nutritional value of adelgid eggs, requiring *S. tsugae* to consume different numbers of eggs of each species to meet its energy requirements.

In the oviposition preference tests, *S. tsugae* females laid eggs on both *A. tsugae* infested hemlock twigs and *A. piceae* infested Fraser fir bark. The predator also demonstrated a preference for ovipositing on prey-infested versus uninfested host plants. These experiments indicate only host acceptance and not that either prey is suitable to stimulate oogenesis in predator females. However, given the very low oviposition rate of *S. tsugae* in these experiments (< 1 egg per female), drawing meaningful conclusions from the data is tenuous.

In both oviposition tests, obtaining accurate predator egg counts proved difficult as the number of newly hatched *S. tsugae* larvae counted in test arenas several days after the trials ended was greater than the number of predator eggs counted. This result is likely due to the fact that *S. tsugae* eggs resemble those of adelgids, and that predator females prefer to oviposit singly in concealed locations under bud scales, in empty seed cones, or within adelgid egg masses (Cheah and McClure 1998). This behavior may partly explain why *S. tsugae* females found *A. piceae* infested Fraser fir suitable for oviposition given the large number of cracks, crevices, lenticels, and lichens typically found on the bark of this tree (Krüssman 1985).

The developmental performance of two *A. tsugae* predators with biological control potential, *Laricobius nigrinus* Fender and *Laricobius* sp. n. (Coleroptera: Derodontidae), has been evaluated on *A. piceae*. Neither species completed egg to adult development, only reaching the fourth larval instar and prepupae stages, respectively, before all individuals died (Zilahi-Balogh et al. 2002; Lamb et al. 2008). In the current study, *S*. *tsugae* successfully completed development on *A. piceae*, doing so at a similar rate as when fed *A. tsugae*. These egg to adult developmental rates, 19.1 (± 0.6) for *A. tsugae* and 18.4 (± 1.2) days for *A. piceae* at 26° C, 16:8 (L:D), and 70–80% RH compare favorably with the previously published *S. tsugae* developmental rate of 17.9 (± 01.0) days at 25° C and 16:8 (L:D) when fed *A. tsugae* in the laboratory (Cheah and McClure 1998).

Although *S. tsugae* successfully completed egg to adult development, the number of individuals surviving each life stage was lower for *A. piceae* versus *A. tsugae* in all stages, except the egg and first larval instar. Lower survival may be the result of differential nutritional quality of each adelgid species for *S. tsugae*, or a lack of prey conditioning as the predators eggs used in this experiment were from *S. tsugae* colonies reared exclusively on a diet of *A. tsugae*. Overall, 60% of beetles survived to the adult stage when fed *A. piceae* compared to 86% when fed *A. tsugae*. By comparison, 17.4% of *L. nigrinus* and 0% of *Laricobius* sp. n. completed egg to adult development when fed *A. tsugae* (Zilahi-Balogh et al. 2002; Lamb et al. 2008).

The importance of supplemental food sources to the survival of adult *S. tsugae* has long been assumed, although its importance has not been well quantified. Mass rearing protocols for the predator call for the regular usage of diluted honey, similar to its use in the experiments reported here, to improve survival of beetles being stored in mass rearing facilities during *A. tsugae's* aestival diapause or when shipped for release (Palmer and Sheppard 2001; Conway et al. 2005). Furthermore, recent research on improved dietary supplements for insectaries and the development of artificial diets for *S. tsugae* has shown that a formulation of egg diet and honey preferentially attracted prolonged adult feeding and supported a mean adult survival rate of 85.1% over a 90 day period (Cohen et al. 2008).

Adult survival results from these experiments indicate that *S. tsugae* longevity over a 36 day period was heavily influenced by the presence or absence of a supplemental food resource, in this case diluted honey. In survival test 1, 84% of predators survived till day 36 when offered diluted honey as the only food resource (food supplement alone treatment). When paired with a prey resource (*A. tsugae* and *A. piceae* plus food supplement treatments), the presence of the supplement increased *S. tsugae* survival from 4 to 96% and 8 to 44% over treatments where the predator was offered either aestivating *A. tsugae* nymphs alone or actively ovipositing *A. piceae* adults alone. Although the presence of diluted honey was less critical in survival test 2 that included actively ovipositing *A. tsugae* adults, *S. tsugae* survival was highest when predators were offered the food supplement alone (88%) and increased from 68 to 76% between the *A. tsugae* alone and *A. tsugae* plus food supplement treatments. The behavioral observations showed that the predator used the food supplement as beetles were found feeding at the diluted honey strip in all treatments where it was included. However, predator behavior was most influenced by the presence or absence of *A. tsugae* infested eastern hemlock twigs where 61.4 to 80.4% of beetles were found when hemlock was present compared to 26.2 and 28.3% of beetles found resting on Fraser fir bark sections.

In the preference and performance tests reported here, *S. tsugae* accepted both *A. tsugae* and *A. piceae* equally for feeding and development suggesting that *A. piceae* might be suitable as an alternate rearing host for the predator. The importance of a supplemental food resource for *S. tsugae* survival during times when prey is scarce or of lower quality was also demonstrated. Additional research is needed to clearly determine if *S. tsugae* females will accept *A. piceae* for oviposition. In the absence of an effective artificial diet, current mass rearing protocols may have to be adjusted to make use of the alternate prey during the aestival diapauses of *A. tsugae*. If *S. tsugae* can be successfully mass reared on *A. piceae* then it might also be possible to expand the biological control potential of the predator to Fraser fir Christmas tree plantations where *A. piceae* is a serious pest issue and its management is currently heavily dependent on insecticides (Potter et al. 2005).

**Table 1.**
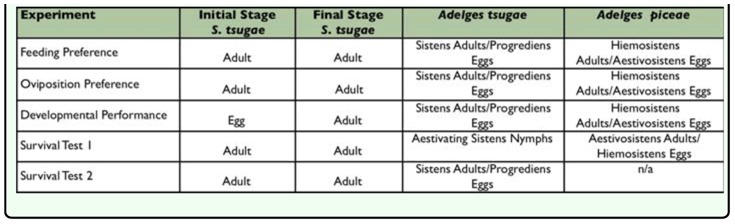
Summary of predator and test prey life stages used in *Sasajiscymnus tsugae* preference, suitability, and survival experiments.

**Table 2.**
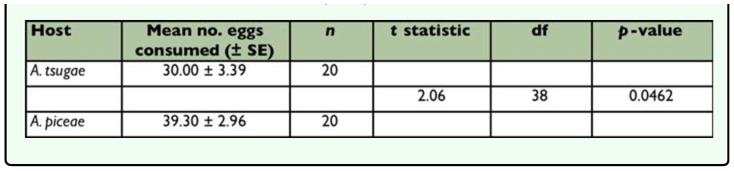
Mean (± SE) number of adelgid eggs consumed by adult *Sasajiscymnus tsugae* in 72-hour feeding rate no choice (single-prey) tests conducted at 26°C, 16:8 (L:D), and 70–80% RH.

**Table 3.**
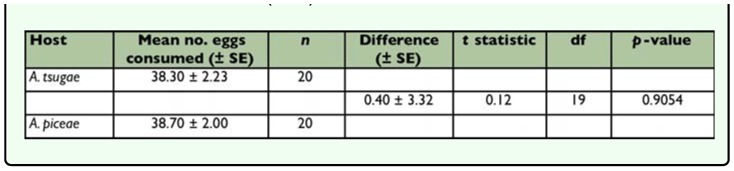
Mean (± SE) number of adelgid eggs consumed by adult *Sasajiscymnus tsugae* in a 72-hour feeding rate paired-choice test conducted at 26°C, 16:8 (L:D), and 70–80 % RH.

**Table 4.**
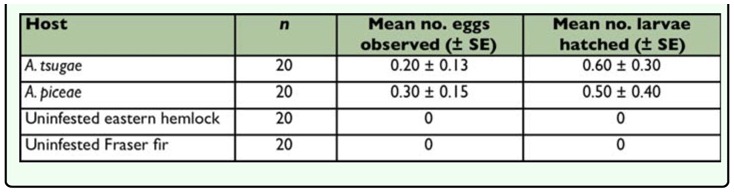
Mean (± SE) number of *Sasajiscymnus tsugae* eggs laid and larvae hatched in a 72-hour paired choice test conducted at 26°C, 16:8 (LD), and 70–80% RH.

**Table 5.**
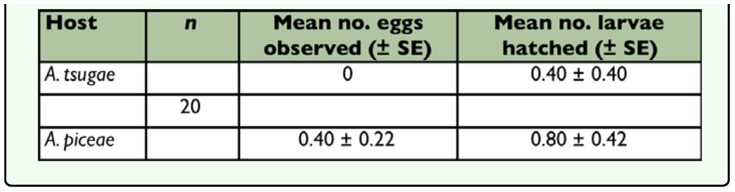
Mean (± SE) number of *Sasajiscymnus tsugae* eggs laid and larvae hatched after the 72-hour paired-choice ovipositional preference test conducted at 26°C, 16:8 (L:D), and 70–80 % RH.

**Table 6.**
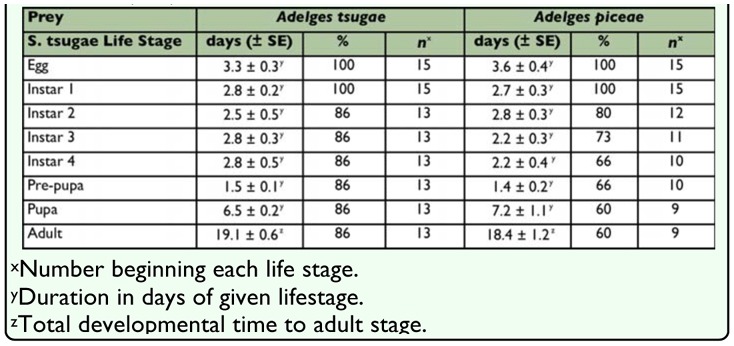
Developmental time (days) and percent (%) survival of *Sasajiscymnus tsugae* from egg to adult stages on two adeglid hosts at 26°C, 16:8 (L:D), and 70–80 % RH.

**Table 7.**
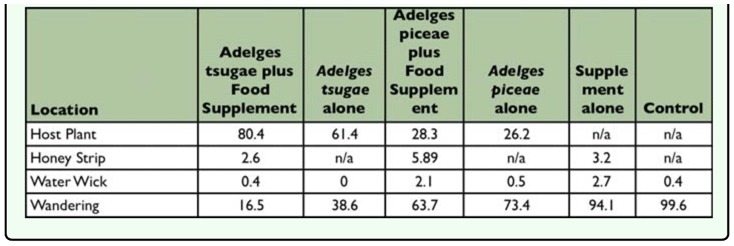
Percentage (%) of live *Sasajiscymnus tsugae* adults found at locations within test arenas in survival test 1.

**Table 8.**
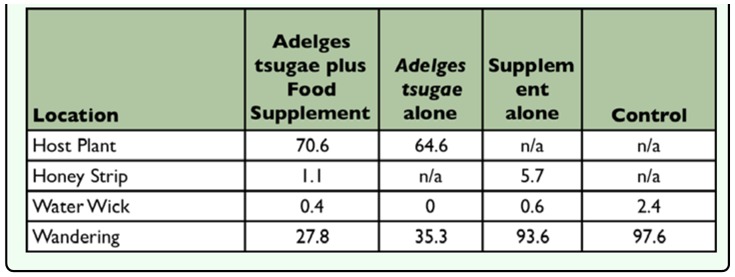
Percentage (%) of live *Sasajiscymnus tsugae* adults found at locations within test arenas in survival test 2.

## References

[bibr01] Annand PN (1924). A new species of *Adelges* (Hemiptera, Phyloxeridae).. *The Pan-Pacific Entomologist*.

[bibr02] Balch RE (1952). Studies of the balsam woolly aphid, *Adelges piceae* (Ratz.) and its effect on balsam fir, *Abies balsamea* (L.) Mill..

[bibr03] Butin EE, Havill NP, Elkinton JS, Montgomery ME (2004). Feeding preference of three lady beetle predators of the hemlock woolly adelgid (Homoptera: Adelgidae).. *Journal of Economic Entomology*.

[bibr04] Cheah CAS-J, McClure MS, Salom SM, Tigner TC, Reardon RC (1996). Exotic natural enemies of *Adelges tsugae* and their prospect of biological control.. *Proceedings of The First Hemlock Woolly Adelgid Review*.

[bibr05] Cheah CAS-J, McClure MS (1998). Life history and development of *Pseudoscymnus tsugae* (Coleoptera: Coccinellidae), a new predator of the hemlock woolly adelgid (Homoptera: Adelgidae).. *Environmental Entomology*.

[bibr06] Cheah CAS-J, McClure MS (2000). Seasonal synchrony of life cycles between the exotic predator, *Pseudoscymnus tsugae* (Coleoptera: Coccinellidae) and its prey, the hemlock woolly adelgid *Adelges tsugae* (Homoptera: Adelgidae).. *Agricultural and Forest Entomology*.

[bibr07] Cheah C, Montgomery ME, Salom S, Parker BL, Costa S, Skinner M (2004). *Biological control of hemlock woolly adelgid*..

[bibr08] Cohen AC, Cheah CAS-J, Strider J, Hain F, Onken B, Reardon R (2008). Diet development for hemlock woolly adelgids and their predators.. *Fourth Symposium on Hemlock Woolly Adelgid in the Eastern United States*.

[bibr09] Conway HE, Burton KB, Hendrix CA, Burgess LW, Cullin JD (2005). Comparison of two different box styles for mass rearing of *Sasajiscymnus tsugae* (Coleoptera: Coccinellidae), a biological control agent of hemlock woolly adelgid (Hemiptera: Adelgidae).. *Canadian Entomologist*.

[bibr10] Dull CW, Ward JD, Brown HD, Bryan GW, Clerke WH, Uhler RJ (1988). *Evaluation of spruce and fir mortality in the Southern Appalachian mountains*..

[bibr11] Havill NP, Montgomery ME, Yu G, Shiyake S, Caccone A (2006). Mitochondrial DNA from hemlock woolly adelgid (Hemiptera: Adelgidae) suggests cryptic speciation and pinpoints the source of the introduction to eastern North America.. *Annals of the Entomological Society of America*.

[bibr12] Jenkins MA (2003). Impact of balsam woolly adelgid (*Adelges piceae* Ratz.) on an *Abies fraseri* (Pursh) Poir. dominated stand near the summit of Mount LeConte, Tennessee.. Castanea.

[bibr13] Krussman G (1985). *Manual of Cultivated Conifers*.

[bibr14] Lamb A, Shiyake S, Salom S, Montgomery M, Kok L, Onken B, Reardon R (2008). Evaluation of the Japanese *Laricobius* sp. n. and other natural enemies of hemlock woolly adelgid in Japan.. *Fourth Symposium on Hemlock Woolly Adelgid in the Eastern United States*.

[bibr15] McClure MS (1987). Biology and control of hemlock woolly adelgid.. *Bulletin of the Connecticut Agricultural Experiment Station*. 851.

[bibr16] McClure MS (1989). Evidence of a polymorphic life cycle in the hemlock woolly adelgid, *Adelges tsugae* (Homoptera: Adelgidae).. *Annals of the Entomological Society of America*.

[bibr17] McClure MS, Salom SM, Shields KS (2003). *Hemlock Woolly Adelgid*..

[bibr18] Mitchell RG, Buffam PE (2001). Patterns of long-term balsam woolly adelgid infestations and effects in Oregon and Washington.. *Western Journal of Applied Forestry*.

[bibr19] Palmer D, Sheppard J (2001). *Notes on rearing Pseudoscymnus tsugae*. Alampi Beneficial Insect Laboratory..

[bibr20] Palmer DJ, Sheppard JL, Onken B, Reardon R, Lashomb J (2002). Mass rearing *Pseudoscymnus tsugae* at the New Jersey Department of Agriculture: Challenges and lessons.. *Symposium on Hemlock Woolly Adelgid in the Eastern United States*.

[bibr21] Potter KM, Frampton J, Sidebottom J, Onken B, Reardon R (2005). Impacts of balsam woolly adelgid on the southern Appalachian spruce-fir ecosystem and the North Carolina Christmas tree industry.. *Third Symposium on Hemlock Woolly Adelgid in the Eastern United States*.

[bibr22] Salom SM, Sharov AA, Mays WT, Neal JW (2001). Evaluation of aestival diapause in hemlock woolly adelgid (Homoptera: Adelgidae).. *Environmental Entomology*.

[bibr23] Salom SM, Kok LT, Lamb A, Jubb C, Onken B, Onken B, Reardon R (2008). Biological control of hemlock woolly adelgid: what is it going to take to make it work.. *Fourth Symposium on Hemlock Woolly Adelgid in the Eastern United States*.

[bibr24] SAS 9.1. (2003). SAS Version 9.1.

[bibr25] Stoetzel MB, Onken B, Reardon R, Lashomb J (2002). History of the introduction of *Adelges tsugae* based on voucher specimens in the Smithsonian Institute National Collect of Insects.. *Symposium on Hemlock Woolly Adelgid in the Eastern United States*.

[bibr26] Wallace MS, Hain FP (2000). Field surveys and evaluation of native and established predators of the hemlock woolly adelgid (Homoptera: Adelgidae) in the southeastern United States.. *Environmental Entomology*.

[bibr27] Young RF, Shields KS, Berlyn GP (1995). Hemlock woolly adelgid (Homoptera: Adelgidae): stylet bundle insertion and feeding sites.. *Annals of the Entomological Society of America*.

[bibr28] Zilahi-Balogh GMG, Kok LT, Salom SM (2002). Host specificity of *Laricobius nigrinus* Fender (Coleoptera: Derodontidae), a potential biological control agent of the hemlock woolly adelgid, *Adelges tsugae* Annand (Homoptera: Adelgidae).. *Biological Control*.

